# Targeted Anti‐Tumor Immunotherapy Using Tumor Infiltrating Cells

**DOI:** 10.1002/advs.202101672

**Published:** 2021-10-18

**Authors:** Yifan Xie, Feng Xie, Lei Zhang, Xiaoxue Zhou, Jun Huang, Fangwei Wang, Jin Jin, Long Zhang, Linghui Zeng, Fangfang Zhou

**Affiliations:** ^1^ School of Medicine Zhejiang University City College Hangzhou 310015 China; ^2^ College of Life Sciences Zhejiang University Hangzhou 310058 China; ^3^ Institutes of Biology and Medical Science Soochow University Suzhou 215123 P. R. China; ^4^ Department of Orthopaedic Surgery The Third Affiliated Hospital of Wenzhou Medical University Rui'an 325200 China; ^5^ MOE Key Laboratory of Biosystems Homeostasis & Protection and Innovation Center for Cell Signaling Network Life Sciences Institute Zhejiang University Hangzhou 310058 China

**Keywords:** anti‐tumor immunity, anti‐tumor therapy, B cells, CD4^+^ T cells, CD8^+^ T cells, T‐cell exhaustion, tumor immunotherapy, tumor microenvironment

## Abstract

In the tumor microenvironment, T cells, B cells, and many other cells play important and distinct roles in anti‐tumor immunotherapy. Although the immune checkpoint blockade and adoptive cell transfer can elicit durable clinical responses, only a few patients benefit from these therapies. Increased understanding of tumor‐infiltrating immune cells can provide novel therapies and drugs that induce a highly specific anti‐tumor immune response to certain groups of patients. Herein, the recent research progress on tumor‐infiltrating B cells and T cells, including CD8^+^ T cells, CD4^+^ T cells, and exhausted T cells and their role in anti‐tumor immunity, is summarized. Moreover, several anti‐tumor therapy approaches are discussed based on different immune cells and their prospects for future applications in cancer treatment.

## Graphical abstract

The progress of anti‐tumor immunity is reviewed. Recent findings of CD4^+^ T cell, CD8^+^ T cell, T cell exhaustion and B cell are provided. Several anti‐tumor immunotherapies based on different immune cells in these years and prospects of anti‐tumor immunotherapies are also discussed. The clinical trials of some immunotherapies are also reviewed.

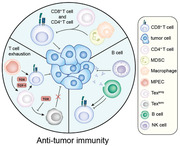

## 1 Introduction

The immunesystem plays an important role in monitoring and clearing tumor cells. However, tumor cells can escape immune monitoring and clearance in a process called immunoediting through a series of mechanisms.^[^ ^1^, ^2^ ^]^ Therefore, treatments that can enhance the anti‐tumor immune response, that is, anti‐tumor immunotherapies, can be used as a new anti‐tumor strategy. Immunotherapy induces long‐lasting anti‐tumor responses and is thus beneficial to the long‐term survival of patients.^[^ ^3^ ^]^ With the development of immune checkpoint blockade and T‐cell genetic engineering therapies, several studies have investigated the metabolic pathways and related immune cells involved in the anti‐tumor response.^[^ ^4^ ^]^

**Figure 1:**
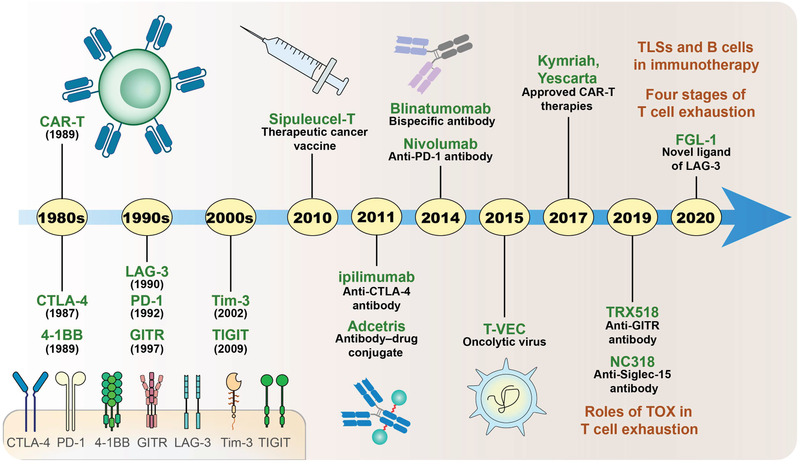
History of cancer immunotherapy. Major breakthroughs on the receptors, strategies, and drugs used for Immunotherapies are indicated.

T cells are of great significance for immunotherapy. Discovered in the 20th century, CTLA‐4 and PD‐1 are the most well‐known targets used in immunotherapies.^[^ ^5^, ^6^, ^7^, ^8^, ^9^ ^]^ Several surface molecules, such as TIGIT, GITR, and LAG‐3, have also been recently found to play special roles in the anti‐tumor immune response and can be used as novel targets (**Figure** **1**).^[^ ^10^, ^11^, ^12^, ^13^ ^]^ The inhibition or activation of these molecules has been demonstrated to exert significant effects on some tumors.

Although there are only a few studies focusing on B cells, they have revealed to play an important role in the tumor microenvironment (TME) (Figure 1).^[^ ^14^, ^15^ ^]^ Thus, anti‐tumor immunotherapies based on B cells may also be a potential anti‐tumor strategy.

Although immunotherapy is an attractive strategy, only a few patients can benefit from it owing to various factors such as low mutation load, immunosuppressive cells, inhibitory receptors, and the depletion of immune cells. Thus, immunosuppressive cells, such as regulatory T (Treg) cells, myeloid derived suppressor cells(MDSCs), and M2‐like macrophages have gained considerable research attention.^[^ ^3^ ^]^ These cells normally play an important role in preventing autoimmunity and immune hyperactivity; however, they have become obstacles in anti‐tumor immunity. Another factor is the depletion of immune cells. Particularly, when effector T (Teff) cells are gradually transformed into depleted T cells during chronic infection or cancer, they lose their effector function. The molecular mechanism of this process has been extensively investigated.^[^ ^16^, ^17^, ^18^ ^]^ Exploring the mechanisms of T‐cell exhaustion may contribute to improving the efficacy of tumor immunotherapies and even the therapies for some chronic infections, such as HIV and hepatitis B.

In this review, we summarize the recent research progress on tumor‐infiltrating T cells and B cells and the mechanism of T‐cell exhaustion. We also discuss some anti‐tumor treatments based on various immune cells and the future prospects of anti‐tumor immunotherapy for cancer treatment.

## 2 Tumor‐Infiltrating T Cells

**Figure 2:**
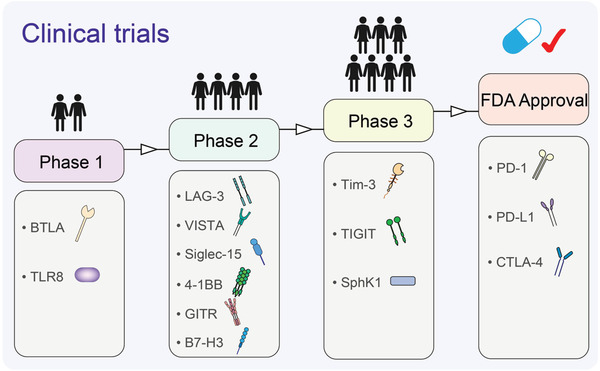
Clinical trials of drugs targeting of the anti‐tumor immune targets. The most progress of clinical transformation of targets mentioned in table 1 are indicated. Some drugs targeting those targets have reached phase 1(BTLA and TLR8), phase 2 (LAG‐3, VISTA, Siglec‐15, 4‐1BB, GITR, and B7‐H3) or phase 3 (Tim‐3, TIGIT, and SphK1) of clinical trials. Some drugs have already received FDA approval (PD‐1, PD‐L1, and CTLA‐4).

T cells are the most important type of cells in anti‐tumor immunity and have multiple populations and functions in the TME. Therefore, they are one of the main target cells in tumor immunotherapy. The well‐known PD‐1/PD‐L1 blockade targets T cells. However, only a few patients benefit from this therapy. In such cases, it is important to understand the mechanism of tumor immunity mediated by T cells to identify new targets. Some drugs that target PD‐1/PD‐L1 and some ongoing clinical trials that use the monoclonal antibodies, antagonists, or agonists of new targets to treat certain cancers are listed in **Table** **1** (Table 1, **Figure** **2**).

**Table 1:** Anti‐tumor immune targets of FDA approved drugs and ongoing clinical trials

Target	Drug name	Type of drug	Phase of trial	Indication	Reference
PD‐1	Nivolumab	Monoclonal antibody	Approved	melanoma, non‐small cell lung cancer, malignant pleural mesothelioma, renal cell carcinoma, classical Hodgkin lymphoma, squamous cell carcinoma of the head and neck, urothelial carcinoma, colorectal cancer, hepatocellular carcinoma, esophageal squamous cell carcinoma	^[^ ^19^ ^]^
	Pembrolizumab	Monoclonal antibody	Approved	melanoma, non‐small cell lung cancer, small cell lung cancer, head and neck squamous cell cancer, classical Hodgkin lymphoma, primary mediastinal large B‐cell lymphoma, urothelial carcinoma, colorectal cancer, gastric cancer, esophageal cancer, cervical cancer, hepatocellular carcinoma, merkel cell carcinoma, renal cell carcinoma, endometrial carcinoma, cutaneous squamous cell carcinoma, triple‐negative breast cancer	
	Cemiplimab	Monoclonal antibody	Approved	metastatic cutaneous squamous cell carcinoma, locally advanced cutaneous squamous cell carcinoma	
	Camrelizumab	Monoclonal antibody	Phase II/III	hepatocellular carcinoma, oesophageal squamous cell carcinoma, classical Hodgkin lymphoma	^[^ ^20^, ^21^, ^22^ ^]^
PD‐L1	Durvalumab	Monoclonal antibody	Approved	locally advanced or metastatic urothelial carcinoma, non‐small cell lung cancer, extensive‐stage small cell lung cancer	^[^ ^19^ ^]^
	Avelumab	Monoclonal antibody	Phase III	renal cell carcinoma, urothelial carcinoma, non‐small‐cell lung cancer	^[^ ^23^, ^24^, ^25^, ^26^ ^]^
	Atezolizumab	Monoclonal antibody	Approved	merkel cell carcinoma, urothelial carcinoma, renal cell carcinoma	^[^ ^19^ ^]^
CTLA‐4	Ipilimumab	Monoclonal antibody	Approved	colorectal cancera, melanoma, renal cell carcinoma	^[^ ^19^ ^]^
LAG‐3	BMS‐986016	Monoclonal antibody	Phase I/II	melanoma	^[^ ^27^, ^28^ ^]^
	LAG525	Monoclonal antibody	Phase II	small cell lung cancer, neuroendocrine tumor, diffuse large B‐cell lymphoma, gastric adenocarcinoma, esophageal adenocarcinoma, castration resistant prostate adenocarcinoma, soft tissue sarcoma, ovarian adenocarcinoma	^[^ ^29^, ^30^ ^]^
	TSR‐033	Monoclonal antibody	Phase I	advanced solid tumors	^[^ ^31^ ^]^
	MK‐4280	Monoclonal antibody	Phase I/II	non‐Hodgkin lymphoma, B‐cell lymphoma	^[^ ^32^ ^]^
	LBL‐007	Monoclonal antibody	Phase I/II	melanoma	N/A
	SHR‐1802	Monoclonal antibody	Phase I/II	malignant tumors	N/A
	sym022	Monoclonal antibody	Phase I	solid tumor, lymphoma	^[^ ^33^ ^]^
	EOC202	Recombinant protein	Phase I	adult solid tumor	^[^ ^32^ ^]^
	IMP321	Recombinant protein	Phase I/II	breast carcinoma, solid tumor	^[^ ^34^, ^35^ ^]^
Tim‐3	TSR022	Monoclonal antibody	Phase II	melanoma	^[^ ^36^ ^]^
	MBG453	Monoclonal antibody	Phase II/III	non‐small cell lung cancer, melanoma, myelodysplastic syndromes, acute myeloid leukemia, chronic leukemia	^[^ ^37^, ^38^ ^]^
	LY3321367	Monoclonal antibody	Phase I	solid tumor	^[^ ^39^ ^]^
	BMS‐986258	Monoclonal antibody	Phase I/II	advanced cancer	^[^ ^32^, ^40^ ^]^
	Sym023	Monoclonal antibody	Phase I	solid tumors, lymphomas	^[^ ^41^ ^]^
	BGBA425	Monoclonal antibody	Phase I	solid tumors	^[^ ^42^ ^]^
	ICAGN02390	Monoclonal antibody	Phase I	solid tumors	^[^ ^43^ ^]^
	SHR‐1702	Monoclonal antibody	Phase I	acute myeloid leukemia, advanced solid tumor	^[^ ^31^ ^]^
	INCAGN2390	Monoclonal antibody	Phase I	advanced malignancies	
TIGIT	MK‐7684	Monoclonal antibody	Phase II	non small cell lung cancer	N/A
	Tiragolumab	Monoclonal antibody	Phase II/III	non‐small cell lung cancer, cervical cancer, small cell lung cancer, blood cancers	^[^ ^44^, ^45^ ^]^
	BMS‐986207	Monoclonal antibody	Phase I/II	advanced solid tumor	^[^ ^31^ ^]^
	AB‐154	Monoclonal antibody	Phase I/II	glioblastoma, non small cell lung cancer, nonsquamous non small cell lung cancer,squamous non small cell lung cancer, lung cancer	
	ASP‐8374	Monoclonal antibody	Phase I	advanced solid tumors	
VISTA	JNJ‐61610588	Monoclonal antibody	Phase I	advanced cancer	^[^ ^46^ ^]^
	CA‐170	Small molecular inhibitor	Phase I/II	solid tumors, lymphomas, non‐small cell lung cancer, prostatic neoplasms	^[^ ^47^, ^48^ ^]^
BTLA	TAB004/JS004	Monoclonal antibody	Phase I	advanced solid tumor, metastatic solid tumor	N/A
Siglec‐15	NC318	Monoclonal antibody	Phase II	head and neck squamous cell carcinoma, non‐small cell lung cancer, ovarian cancer, triple negative breast cancer	^[^ ^49^ ^]^
SphK1	Icaritin	Small molecular inhibitor	Phase III	hepatocellular carcinoma	^[^ ^50^ ^]^
4‐1BB	Urelumab	Monoclonal antibody	Phase I/II	solid tumors, B‐cell non‐Hodgkin's lymphoma	^[^ ^51^ ^]^
	Utomilumab	Monoclonal antibody	Phase I/II	oropharyngeal cancer, large B‐cell lymphoma, breast cancer	^[^ ^52^ ^]^
GITR	MEDI1873	Recombinant protein	Phase I	advanced solid tumors	^[^ ^53^ ^]^
	AMG 228	Monoclonal antibody	Phase I	advanced solid tumors	^[^ ^54^ ^]^
	BMS‐986156	Monoclonal antibody	Phase I/II	solid tumors	^[^ ^55^ ^]^
	TRX518	Monoclonal antibody	Phase I/II	melanoma, triple negative breast cancer, metastatic castration‐resistant prostate cancer, platinum‐resistant ovarian cancer	^[^ ^56^, ^57^ ^]^
	ASP1951	Monoclonal antibody	Phase I	advanced solid tumors	^[^ ^57^ ^]^
	INCAGN01876	Monoclonal antibody	Phase I/II	metastatic cancer, glioblastoma, cancer of the head and neck	^[^ ^58^ ^]^
	GWN323	Monoclonal antibody	Phase I	solid tumors, lymphomas	
	MK‐4166	Monoclonal antibody	Phase I	advanced solid tumors	^[^ ^59^ ^]^
TLR8	DN1508052	Agonist	Phase I	solid tumors	N/A
TLR7/8	Motolimod	Agonist	Phase I	squamous cell carcinoma of the head and neck	^[^ ^60^ ^]^
	NKTR‐262	Agonist	Phase I/II	melanoma, merkel cell carcinoma, triple negative breast cancer, head and neck squamous cell carcinoma, renal cell carcinoma, colorectal cancer, sarcoma	^[^ ^61^ ^]^
NRP‐1	ASP1488	Monoclonal antibody	Phase I	advanced solid tumors	^[^ ^62^ ^]^
B7‐H3	Énoblituzumab	Monoclonal antibody	Phase II	head and neck cancer, prostate cancer	^[^ ^63^ ^]^
	I‐8H9	Monoclonal antibody	Phase I	neuroblastoma	^[^ ^64^ ^]^

### 2.1 Anti‐Tumor Targets Associated with CD8^+^ T Cells

**Figure 3:**
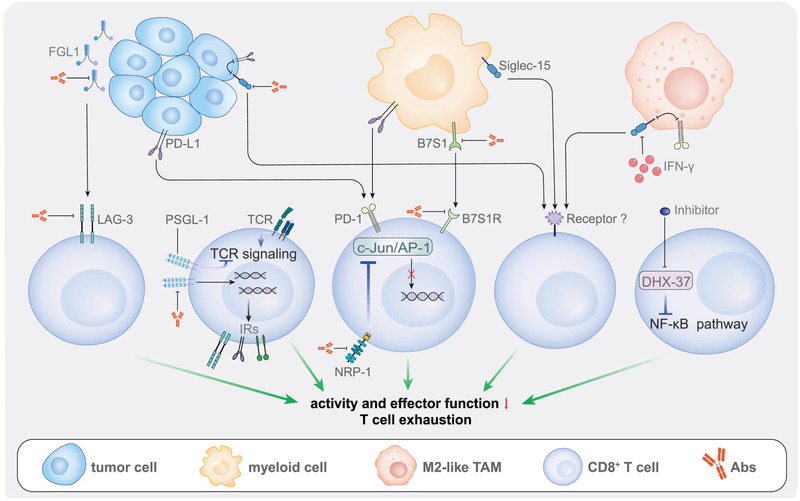
Anti‐tumor targets associated with CD8^+^ T cells. These targets include co‐inhibitors on the surface of immune cells or intracellular proteins affecting the activity of CD8^+^ T cells. The blockade of these targets can lead to more effective therapeutic effects.

CD8^+^ T cells constitute an important population of tumor‐infiltrating lymphocytes (TILs). Effector CD8^+^ T cells can kill tumor cells and secrete several kinds of cytokines, such as IFN‐*γ* and IL‐2. However, to avoid clearance, tumor cells have developed the ability to inhibit the effector function of tumor‐infiltrating CD8^+^ T cells (**Figure** **3**). Researchers have investigated the mechanism by which tumor cells escape clearance mediated by CD8^+^ T cells and strategies to block these mechanisms.

Co‐inhibitory checkpoint receptors are one of the most important reasons of immunosuppression in TMEs. For instance, LAG‐3 is an important inhibitory receptor that is highly expressed on depleted CD8^+^ T cells, CD4^+^ T cells, and NK cells and can inhibit the proliferation and effector activity of these cells.^[^ ^12^, ^65^, ^66^ ^]^ Recently, Jun Wang et al. discovered FGL‐1, an important ligand of LAG‐3,^[^ ^67^ ^]^ that is normally released by liver cells but is also highly expressed by tumor cells. The knockout of its gene increased the number of CD8^+^ T cells and effectively curbed the development of tumors, suggesting that the high expression of FGL‐1 in tumor cells may be an important mechanism by which tumors escape immune clearance and may contribute to the poor patient response to PD‐1 blockade therapies.

NRP‐1 and B7S1 are two important molecules in the system of co‐inhibitory checkpoint, which play important roles in the immune system, and are also related to the function and activity of CD8+ T cells. NRP‐1 is expressed in T cells and dendritic cells (DCs) and is related to their interaction.^[^ ^68^, ^69^, ^70^ ^]^ However, NRP‐1 also maintains Treg survival and inhibits CD8^+^ T cells during tumorigenesis, which eventually hinders tumor clearance mediated by effector T cells.^[^ ^68^, ^69^, ^70^ ^]^ Its role in tumorigenesis and its potential as a drug target have been confirmed.^[^ ^71^, ^72^, ^73^ ^]^ However, the specific mechanism by which NRP‐1 affect tumorigenesis remains unknown. Chang Liu et al. found that NRP‐1 receptors on CD8^+^ T cells inhibit the expression of c‐Jun/AP‐1 after binding with ligands, leading to memory T (Tmem) cell differentiation and CD8^+^ T cell depletion, thus ultimately reducing the number of tumor‐infiltrating CD8^+^ T cells.^[^ ^74^ ^]^ Therefore, blocking NRP‐1 can increase the number of tumor‐infiltrating CD8^+^ T cells to provide a suitable environment for immunotherapy, such as PD‐1 blockade. Meanwhile, B7S1, an important member of the B7 family, is a negative immune regulator expressed on myeloid cells.^[^ ^75^ ^]^ Li et al. suggested that B7S1 may induce the depletion of CD8^+^ T cells via the overexpression of Eomes and B7S1. They also found that PD‐1 has joint effects in inhibiting CD8^+^ T cell function in mice.^[^ ^76^ ^]^ Therefore, simultaneously blocking B7S1 and PD‐1 may be a potential immunotherapy strategy for tumors.

Other proteins related to co‐inhibitory checkpoint pathways affecting the anti‐tumor activity of CD8^+^ T cells have also been identified. For example, blocking NKG2A, which plays a role in immunosuppression, exhibited a significant anti‐tumor effect by enhancing the anti‐tumor response of CD8^+^ T cells rather than NK cells. NKG2A is suggested as the “culprit” of the adaptive tolerance of CD8^+^ T cells to cancer vaccines.^[^ ^77^ ^]^ The upregulation of siglec‐15, an important immunosuppressive factor, was detected in tumor cells, myeloid cells, and M2‐like macrophages in the TME and was mutually exclusive with PD‐L1 expression, which may be related to some cytokines such as M‐CSF and IFN‐*γ*. Gene digestion and antibody blocking of siglec‐15 in mouse models resulted in tumor inhibition.^[^ ^78^ ^]^ The promising potential of the siglec–sialidan axis as drug target has been recently reported.^[^ ^79^ ^]^

Beside proteins related to co‐inhibit checkpoint pathway, other intracellular or extracellular protein may control the existence of CD8+ T cell in TMEs. PSGL‐1, a cell‐adhesion molecule on T cells, mediates selectin‐dependent leukocyte adhesion and is closely related to inflammatory reactions.^[^ ^80^ ^]^ Recent studies have shown that it inhibits TCR and IL‐2 signals, upregulates PD‐1 expression in depleted CD8^+^ T cells, and inhibits the effector function of CD8^+^ T cells.^[^ ^81^ ^]^ In addition, PSGL‐1 is a receptor of the important anti‐tumor target protein VISTA. The blockade of both has shown certain anti‐tumor effects.^[^ ^82^, ^83^ ^]^ In 2019, a genome‐wide CRISPR screening technology identified the novel targets associated with anti‐tumor immunity. Particularly, the RNA helicase DHX37, which regulates the NF‐*κ*B signaling pathway, was revealed to inhibit multiple functions of tumor‐infiltrating CD8+ T cells. Thus, this enzyme is a potential target in anti‐tumor immunotherapy.^[^ ^84^ ^]^ Moreover, the composition and behavior of CD8^+^ T cells in the TME have attracted research attention. To elucidate the possible anti‐tumor mechanism of PD‐1 therapy using single‐cell RNA sequencing, Yost et al. found that PD‐1 blockade promotes the replacement of depleted T cells in the TME using CD8^+^ T cell clones from outside the tumor.^[^ ^85^ ^]^ Wu et al. further demonstrated that CD8^+^ T cells around the tumor and not those in the TMEs play the most essential role in checkpoint blockade therapies.^[^ ^86^ ^]^ Therefore, the ability of T cells around the tumor to enter the tumor area may be a key in determining the effect of anti‐tumor immunotherapies. In addition, Dangaj et al. showed that the recruitment of T cells into the TME requires chemokines such as CCL5 secreted by tumor cells, CXCL9 secreted by myeloid cells and IFN‐*γ*‐inducible. Epigenetic silencing of CCL5 may be an immune escape mechanism of tumors that inhibits the entry of CD8+ T cells into the TME and forced CCL5 expression prevented tumor‐infiltrating lymphocyte desertification, which indicated that recovering the expression of CCL5 in tumors may be a key of anti‐tumor immunotherapies.^[^ ^87^ ^]^ Some important CD8^+^ T cell subsets in the TME, including stem‐like CD8^+^ T cells, can differentiate into effector CD8^+^ T cells in the suitable environment of the TME, thus serving as sources of CD8^+^ T cells responsive to ICT^[^ ^88^ ^]^ and bystander CD8^+^ T cells lacking CD39 proteins.^[^ ^89^ ^]^ Therefore, the infiltration of these cells into the TME may be closely related to the efficacy of anti‐tumor immunotherapies.

### 2.2 Tumor‐Infiltrating CD4^+^ T Cells in the TME

**Figure 4:**
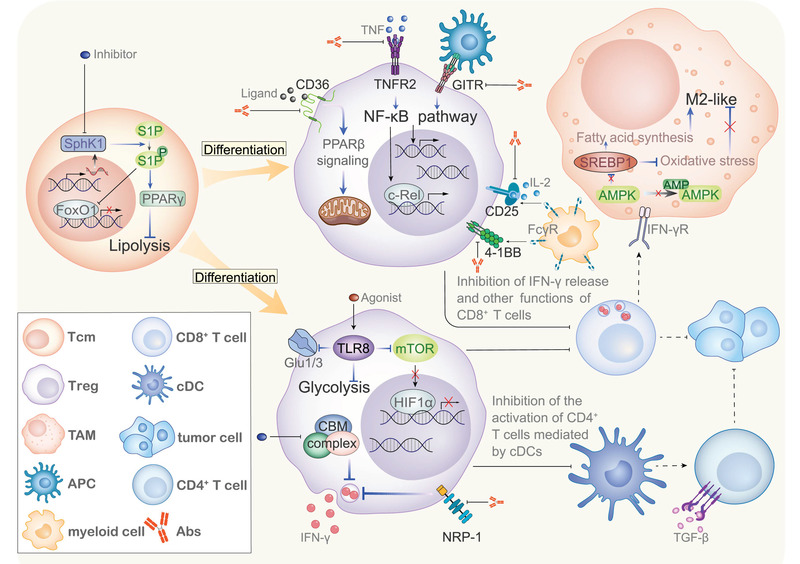
Tumor‐infiltrating CD4^+^ T cells in the TME. The roles of CD4^+^ T cells, especially Treg cells, in the TME have received increasing research attention. Several receptors and intracellular proteins that affect the differentiation and the immunosuppressive function of T_reg_ cells have been identified, many of which can become potential targets. Treg cells not only directly inhibit the activity of CD8^+^ T cells, but also exert immunosuppressive functions via tumor‐associated macrophages (TAMs) and other CD4^+^ T cells.

CD4^+^ T cells are another type of important T cell in the TME. There are many cells involved in the population of CD4^+^ T cells, including T helper type 1 cells , T helper type 2 cells, T helper type 17 cells, and Treg cells. These cells have different functions in the immune system, such as secretion of several kinds of cytokines and assisting in antigen presentation. In tumor immunity, some CD4^+^ T cells enhance the anti‐tumor activity of cytotoxic T cells (CTLs) by promoting their infiltration of tumor sites, maintaining their activities, and performing antigen presentation.^[^ ^90^, ^91^ ^]^ The activation of CD4^+^ T cells that recognize MHC‐II molecules is also crucial for anti‐tumor immunity.^[^ ^92^, ^93^ ^]^ However, Treg cells suppress effector T cells in tumor immunity. Normally, they help prevent autoimmunity or excessive immune response; however, in the TME, this immunosuppressive effect becomes a huge obstacle to anti‐tumor immunity (**Figure** **4**).^[^ ^94^, ^95^ ^]^ Therefore, the inhibition of the immunosuppressive activity of tumor‐infiltrating T_reg_ cells has become a hotspot in tumor immunotherapy research.

Signaling pathways related to cell metabolism determined the existence of Treg in TMEs, which means molecule in these pathways may be targets to relieve the immunosuppression in TMEs and to enhance the efficiency of anti‐tumor therapies. Sphingosine‐1‐phosphate (S1P) is a lipid molecule that regulates various physiological processes and is closely related to angiogenesis and T cell migration^[^ ^96^ ^]^ and is associated with the occurrence of several cancers.^[^ ^97^ ^]^ Active S1P directly inhibited the expression of FoxO1 proteins and lipolysis by activating the lipid transcription factor PPAR*γ*, which suppresses the mitochondrial function and promotes the differentiation of center memory T (Tcm) cells to Treg cells. Furthermore, blocking SphK1, which activates S1P and catalyzes PPAR*γ* synthesis, led to a certain anti‐tumor response in mouse models.^[^ ^98^ ^]^ Therefore, the SphK1‐S1P pathway blockade may be a potential cancer treatment.

The fatty acid receptor CD36 promotes tumor cell metastasis and is a universal biomarker for metastatic tumor cells.^[^ ^99^ ^]^ Wang et al. reported that CD36 can regulate the adaptive ability of the mitochondria through the PPAR*β* pathway, leading to Treg adaptation in the TME with high levels of lactic acid.^[^ ^100^ ^]^ A recent research showed that CD36 also reduces tumor‐infiltrating CD8+ T cells by mediating the process of ferroptosis.^[^ ^101^ ^]^ Because of the its existence in many cells, using tumor‐specific carriers with CD36 inhibitor or antibody or bispecific antibodies to inhibit CD36 may be a potential anti‐tumor strategy. Actually, clinical trials using bispecific antibodies have achieved certain results.^[^ ^102^ ^]^ Thus, the CD36 blockade may be a potential therapeutic strategy to reduce tumor‐infiltrating Treg cells and promote anti‐tumor immunity. The activation of TLR8, one of the most highly expressed toll‐like proteins in innate immunity, reverses Treg function.^[^ ^103^ ^]^ In 2019, Li et al. reported that TLR8 could reverse the inhibitory effect of Treg cells on Teff cells in the TME by inhibiting glucose uptake, glycolysis, and the mTOR‐HIF1*α*‐axis,^[^ ^104^ ^]^ thereby indicating the potential of TLR8 agonists as effective anti‐tumor drugs, and some drugs based on this strategy are on their own.

The NF‐*κ*B signaling pathway is closely related to the function of Treg cells. Particularly, c‐Rel, an important component of the NF‐*κ*B pathway, plays a critical role in the maintenance of the survival of activated Treg cells .^[^ ^105^ ^]^ Li et al. reported that c‐Rel promotes the production of MDSCs,^[^ ^106^ ^]^ suggesting its potential as anti‐tumor target for drug development. Another receptor closely related to NF‐*κ*B, TNFR2 promotes cell survival and proliferation. Torrey et al. observed the effective anti‐tumor activity of TNFR2 antibodies and found that TNFR2 is highly expressed in Tregs in the TME but not in other T_reg_ cells in vivo,^[^ ^107^ ^]^ indicating that TNFR2 inhibition is promising tumor‐specific antitumor strategy that does not induce strong autoimmunity. GITR is also a widely used target that can activate the NF‐*κ*B pathway via TNF receptor‐associated factors (TRAFs) and is highly expressed in tumor‐infiltrating CD8^+^ T cells and CD4^+^ T cells. Combined treatment with GITR and PD‐1 effectively inhibited tumor‐infiltrating T_reg_ cells and enhanced the effector function of CD8^+^ T cells.^[^ ^56^, ^108^ ^]^

IFN‐*γ* is a cytokine that is widely involved in the induction of Antigen‐presenting cells (APCs) to express histocompatibility antigens (MHC I/II) and activate macrophages. Grasso et al. proposed that decreased IFN‐*γ* expression contributes to tolerance to checkpoint blockade therapies.^[^ ^109^ ^]^ Overacre delgoffe et al. reported that IFN‐*γ* induces T_reg_ cells to be vulnerable to the TME, thus reducing the inhibition of effector T cells and enhancing anti‐tumor response.^[^ ^110^ ^]^ Treg cells can also inhibit the IFN‐*γ* secretion activity of CD8^+^ T cells to activate the differentiation of M2‐like macrophages and subsequently suppress the activity of effector cells in the TME.^[^ ^111^ ^]^ Because of the anti‐tumor effects of IFN‐*γ*, the strategy of promoting their secretion using tumor‐infiltrating immune cells has been explored. Knocking out NRP‐1, an important T cell surface molecule, or reducing CARMA1 expression in Treg cells to destroy the CARMA1‐BCL10‐MALT1 (CBM) complex effectively promoted IFN‐*γ* secretion by T_reg_ cells^[^ ^110^, ^112^ ^]^ and led to an effective anti‐tumor response. Thus, IFN‐*γ*‐related proteins may show promising potential in the development of anti‐tumor targeted drugs.

Other potential targets of Treg cells have also attracted the attention of researchers. For instance, 4‐1BB is an important co‐activated receptor expressed in CD8^+^ T cells and Treg cells. Monoclonal antibodies against 4‐1BB shown effective tumor clearance.^[^ ^113^, ^114^ ^]^ Similar to 4‐1BB, CD25, which is a highly expressed marker on Treg cells, has functions related to Fc*γ*R in myeloid cells. Vargas et al. proposed that the upregulation of the myeloid cell inhibitor Fc*γ*R in the TME contributes to the failure of CD25 antibodies to achieve a significant anti‐tumor effect. Subsequently, they utilized a new type of anti‐CD25 antibodies, which have stronger binding abilities to activate Fc*γ*Rs, and combined them with PD‐1 blockade to deplete T_reg_ cells to obtain satisfactory anti‐tumor effects.^[^ ^115^ ^]^ Furthermore, blocking the IL‐2 signal on effector T cells using anti‐CD25 antibodies can limit the effect of these antibodies. Thus, novel anti‐CD25^NIB^ antibodies that do not affect IL‐2 signaling were developed, and good results following monotherapy or its combination with PD‐1 blockade were achieved.^[^ ^116^ ^]^ Further, RG6292, a new therapeutic antibody with the same effect, was evaluated at the pre‐clinical stage and exerted great effects without overt immune‐related toxicities in both humanized mice and cynomolgus.

Instead of inhibiting CD8^+^ T cells and affect anti‐tumor immunity, T_reg_ cells may also inhibit other tumor‐infiltrating effector cells. Binewies et al. revealed that Treg cells inhibit the response of effector CD4^+^ T cells, conventional proinflammatory T (T_conv_) cells by inhibiting conventional type 2 dendritic cells (cDCs).^[^ ^117^ ^]^ This discovery highlights the role of cDCs in the initiation of the anti‐tumor response mediated by T_conv_, which may become a novel hotspot in anti‐tumor immunity studies.

## 3 T‐Cell Exhaustion: Stages and Related Proteins

Exhausted T (Tex) cells are a special population of differentiated and dysfunctional T cells in the process of chronic infection and tumorigenesis. These cells are derived from memory precursor effector cells (MPECs) rather than effector T cells and have a special differentiation program.^[^ ^118^ ^]^ Tex adapts to continuous antigen stimulation and plays a certain effect function albeit weak without causing immunopathological reactions.^[^ ^119^, ^120^, ^121^ ^]^ However, due to the existence and dysfunction of Tex cells, long‐term immunity to some viruses cannot be established,^[^ ^122^, ^123^ ^]^ and tumor immunotherapies are greatly limited. Therefore, understanding the mechanism of T cell exhaustion is indispensable for anti‐tumor immunity studies.

The long‐term existence of antigens is the main reason for T cell exhaustion. However, the mechanism underlying this process remains unclear. One of the essential elements of T cell exhaustion is a continuous T cell signal, which activates many trigger molecules in different pathways, such as IRF4, PTPN2, and Blimp‐1.^[^ ^124^, ^125^ ^]^ Certain cytokines also play an important role in T cell exhaustion. For instance, Liu et al. found that IL‐2 is an inducer of exhaustion as an environmental cue by activating STAT5.^[^ ^126^ ^]^ IL‐10 and IL‐35 that are normally released by Treg cells and have partially overlapping but non‐redundant functions also induce exhaustion.^[^ ^127^ ^]^

The mitochondria generate most of the energy used by cells but also produce reactive oxygen species (ROS), which may harm the cells. Recently, the relationship between mitochondrial stress and T cell exhaustion has been revealed.^[^ ^128^, ^129^ ^]^ Therefore, mitochondria‐targeted antioxidants may be helpful in resisting T cell exhaustion. Notably, their effects have been demonstrated in anti‐viral treatments and anti‐tumor immunotherapies.^[^ ^130^, ^131^, ^132^ ^]^ Other factors, such as immunosuppressive cells and metabolic changes, are also important in T cell exhaustion. In 2019, Ma et al. found that cholesterol can induce T cell exhaustion through the ER stress sensor XBP1,^[^ ^133^ ^]^ suggesting the existence of other pathways related to T cell exhaustion.

**Figure 5:**
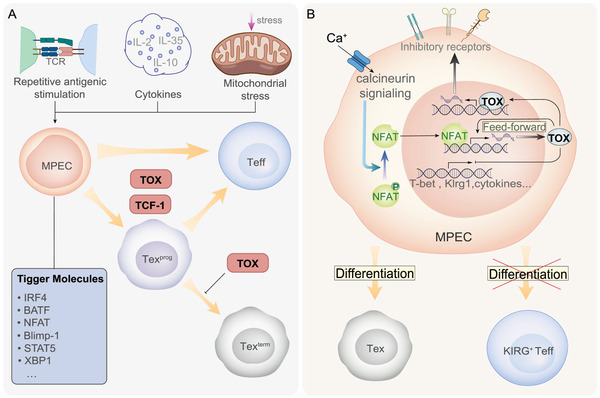
Stages and mechanism of T cell exhaustion. A| The four stages of T cell exhaustion are controlled by a series of proteins, the mechanism of which is complex and involves several environmental factors and trigger molecules. B| Roles of TOX in CD8^+^ T cell exhaustion. TOX, one of the most important proteins during T cell exhaustion, regulates the expression of a series of genes and determines the direction of cell differentiation.

T_ex_ is a heterogeneous cell population, and many protein factors play distinct but interconnected roles during T cell exhaustion (**Figure** **5**). In terms of differentiation, T_ex_ mainly includes two populations: progenitor‐exhausted T cells and terminally exhausted T cells.^[^ ^119^ ^]^ Progenitor‐exhausted cells T cells are essential in tumor ICB therapies, and preventing the differentiation of progenitor exhausted T cells into terminally exhausted T cells is important for tumor immunotherapies,^[^ ^121^, ^134^ ^]^ indicating that progenitor exhausted cells may be essential in successful anti‐tumor immunotherapies. Recently, Beltra et al. summarized the four developmental stages of Tex cells, namely Tex^prog1^, Tex^prog2^, Tex^int^, and Tex^term^ (Figure 1),^[^ ^135^ ^]^ and reported that the function of some proteins are important in T_ex_ development (Figure 5A). In addition to revealing the function of Eomes and TCF‐1, which have been widely studied, Beltra et al. showed the opposite function of T‐bet proteins, which promote the further differentiation of Tex^prog2^ into Tex^int^ but inhibits the appearance of the terminally exhausted phenotype. Moreover, this study emphasized the important role of TOX protein in T cell exhaustion, which has been verified by other studies (Figure 1 and Figure 5B).^[^ ^136^, ^137^, ^138^, ^139^ ^]^ Surprisingly, the differentiation from memory cells to progenitor exhausted cells induced by TOX proteins prevents memory cells from differentiating into Teff cells and is rapidly lost. Therefore, Tex cells should be redefined, and targets related to reversing the appearance of the terminally exhausted phenotype should be explored. Recently, Si et al. reported that HPK‐1 can activate BLIMP1 through the NF‐*κ*B pathway to induce T cell exhaustion.^[^ ^140^ ^]^ This finding indicates our limited understanding of mechanism of T cell exhaustion, and future studies should be more comprehensive.

Although the core features of exhausted T cells induced by chronic infections are consistent with those of Tex cells caused by tumors, Tex cells in the TME also exhibits heterogeneity within and among tumors due to the multiple and complex components in the TME.^[^ ^141^ ^]^ Because of the heterogeneity of Tex cells, it is thus necessary to detect Tex cells in different TMEs at the single‐cell level for personalized cancer treatment. Tirosh et al. and Li et al. analyzed the TME of melanoma using single‐cell RNA‐seq and other technologies and advanced the understanding of the heterogeneity of Tex cells.^[^ ^142^, ^143^ ^]^ Li et al. reported that normal CD8^+^ T cells and Tex cells in the TME of melanoma are continuous and highly dynamic cell populations and detected a discrete toxic CD8^+^ T cell population. In 2018, Bengsch et al. developed a method based on epigenetic and transcriptomic analysis to monitor the heterogeneity of Tex cells,^[^ ^144^ ^]^ which is of great significance for personalized treatment strategies for Tex cells in different TME.

## 4 B Cells in the TME

**Figure 6:**
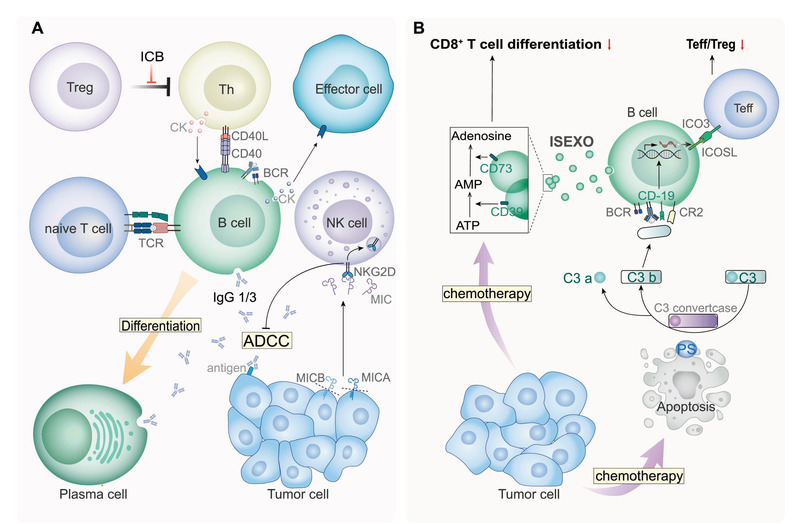
B cells in the TME. A| Functions of B cells in anti‐tumor immunity. B cells various functions in the TME, including cytokine release, antibody production, antigen presentation, and ADCC. Thus, B cells have promising potential as targets for anti‐tumor therapy strategies. B| Role of B cells in chemotherapy. On the one hand, B cells inhibit the differentiation of CD8^+^ T cells by secreting exosomes and hydrolyze the ATP released by tumor cells into adenosine. On the other hand, B cells increase the ratio of Teff/Treg through the complement system, ultimately enhancing anti‐tumor immunity.

T cells, NK cells, DCs, macrophages, and other cells in the TME have been extensively investigated; however, only a few studies have focused on the role of B cells, another important group of immune cells in the TME (**Figure** **6**A). The functions of B cells in colon cancers have been related to follicular helper T (Tfh) cells.^[^ ^145^ ^]^ Hollern et al. have shown that Tfh cells respond to ICB therapies by activating B cells in breast cancers.^[^ ^146^ ^]^ Wieland et al. identified specially activated germinal center B cells and plasma cells in the TME of head and neck cancers.^[^ ^147^ ^]^ In addition, B cell receptors (BCRs), which are closely related to some blood cancers, may also play an important role in solid tumors.^[^ ^148^ ^]^ A recent review article discussed the various roles of different B cell subunits in the TME.^[^ ^15^ ^]^ In 2019, Hu et al. revealed that B cells induce the ADCC reaction of NK cells and exhibit anti‐tumor function by releasing certain antibodies. However, they also found that the copy number of MICA and MICB, as well as the expression of metalloproteinase, increased in the TME with high B‐cell activity, which may be a mechanism by which tumor cells adapt to B‐cell‐mediated anti‐tumor immune response.^[^ ^149^ ^]^ Therefore, understanding the role of B cells in tumorigenesis may become a novel hotspot in tumor research.

Tertiary lymphoid structures (TILs) are ectopic lymphoid organs that are important for anti‐tumor immunity and anti‐chronic infection. Their presence at the tumor site is associated with better clinical response.^[^ ^150^ ^]^ Recently, B cell infiltration and TILs have been positively correlated with ICB success (Figure 1).^[^ ^151^, ^152^, ^153^ ^]^ Therefore, activating B cell infiltration and TIL generation may be novel strategies for future anti‐tumor immunotherapies.

B cells also play dynamic or even opposite roles in the TME after chemotherapy (Figure 6B). A recent study showed that phosphatidylserine (PS) molecules on the surface of apoptotic cells induced by chemotherapy can activate complement signaling and activate the production of some subunits of B cells that do not produce IL‐10 but express ICOL, thus activating T_eff_ and exerting effective antitumor response.^[^ ^154^ ^]^ In addition, the hypoxia inducible factor‐1*α* (HIF‐1*α*) in B cells can induce Rab27A transcription, which leads to the release of CD19^+^ exosomal vesicles by B cells. These vesicles hydrolyze the ATP released by tumor cells into adenosine and subsequently inhibit the anti‐tumor response mediated by CD8^+^ T cells through these adenosines.^[^ ^155^ ^]^ Therefore, HIF‐1*α* and Rab27A may be novel targets for the development of anti‐tumor drugs.

## 5 Overview of Cancer Therapies and Other Tumor‐Related Immune Cells

**Figure 7:**
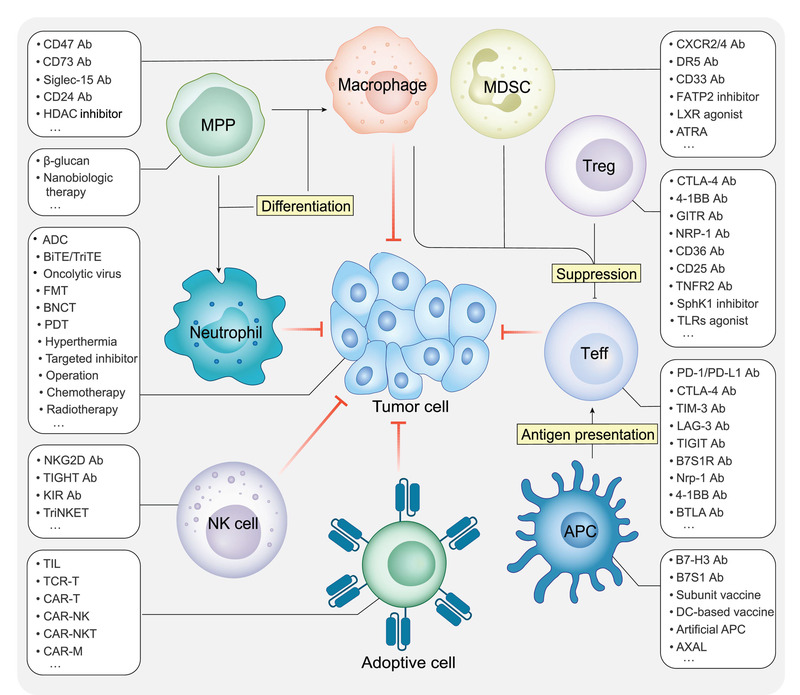
Overview of cancer therapies and other tumor‐related immune cells. In addition to traditional cancer treatment methods, such as surgical resection, radiotherapy, and chemotherapy, new therapies, such as hyperthermia, PDT and immunotherapy, have emerged. Immunotherapy, the most popular alternative treatment to treat tumors, has many strategies. T cells, as well as many other cell types, have become targets of immunotherapy strategies.

Cancer poses a huge threat to human health; therefore, scientists and medical workers work hard to develop novel strategies for cancer treatment and improve the efficacies of current methods. Traditional cancer treatment, including surgical resection, radiotherapy, and chemotherapy, has various limitations. Chemotherapy and radiotherapy can cause serious damage to healthy cells. Therefore, it is necessary to identify novel tumor treatment methods with fewer side effects and higher efficacy. Presently, tumor hyperthermia, photodynamic therapy, irreversible electroporation, local cryotherapy, and immunotherapy, the most popular one, have emerged as alternative cancer treatment methods (**Figure** **7**).^[^ ^156^ ^]^ Many refractory or common tumors are expected to be treated by these novel methods.

### 5.1 Photodynamic Therapy, Boron Neutron Capture Therapy, and Nanodrugs

To reduce the damage to normal cells, tumor‐specific therapy that can precisely target tumors, including well‐known targeted drug therapy, has become an urgent need. Photodynamic therapy (PDT) based on photochemical reactions and boron neutron capture therapy (BNCT) based on thermal neutron nuclear reaction are two important targeted therapies, PDT have already been approved by FDA and BNCT have been approved by the Japanese Ministry of Health, Labour and Welfare as therapies for certain cancer.^[^ ^157^, ^158^ ^]^ Recently, Ravetz et al. used low‐energy near‐infrared light as light source to address the low PDT penetration rate and the competition between the photochemical substrate and the photosensitizer in absorbing incident light.^[^ ^159^ ^]^ Besides, a group of scientists reported a supramolecular photocatalyst, NanoSA‐TCPP, and got great anti‐tumor effect in mice.^[^ ^157^ ^]^ In 2017, Kuthala et al. developed a nanodrug (10bsgrf) for BNCT NPs that can cross the blood–brain barrier and target glioblastoma multiforme successfully in mouse model.^[^ ^160^ ^]^ These advances further confirm the excellent potential of these two tumor therapies, however, more clinical trials should be done to test the effect of those drugs in patients.

With the development of nano materials, nanoparticles have been widely used as to deliver drugs and antigens or to engineer immune cells in many anti‐tumor therapies.^[^ ^161^, ^162^, ^163^ ^]^ There has been several nanodrugs, like Doxil and Abraxane, approved by FDA and many other nanodrugs have entered different stages of clinical trials.^[^ ^164^ ^]^ Recently, Sindhwani et al found that nanoparticles transport through inter‐endothelial gaps in a positive way.^[^ ^165^ ^]^ Li, et al. reported that autonomous RAS signaling and IGF1R kinase in cancer cells play important role in the uptake of Nanoparticulate albumin bound paclitaxel, a widely used anti‐tumor nanodrug.^[^ ^166^ ^]^ These discoveries gave us better understanding of nanodrugs and is beneficial for us to enhance the efficacy of nanodrugs. In these years, lots of researchers reported nanodrugs based on different mechanisms they designed. For example, Zhao, et al. designed a nanocatalyst to realized combined hole/hydrogen therapy, Li , et al. encapsulated self‐replicating RNA in lipid nanoparticles (LNP) and observed anti‐tumor effect in several mouse models of cancer.^[^ ^167^, ^168^ ^]^ However, just as what Sun, et al. mentioned, there may be misunderstanding in the development of nanodrugs.^[^ ^169^ ^]^ Therefore, we need more researches focus on changes of TMEs after drug administration and we need to be very cautious before the success of clinical trials. It is worth mentioning that other biomaterials such as artificial APC and drug delivery system based on biomaterials have also made great progress in anti‐tumor treatment.^[^ ^161^ ^]^

### 5.2 Overview of Anti‐Tumor Immunotherapies

Tumor immunotherapy is one of the most popular tumor treatments today. It activates the immune system to promote immune monitoring and clearance of tumor cells, thereby eliminating tumors. In addition to the well‐known checkpoint blocking therapy (ICT), and adoptive cell transfer therapy, other strategies based on anti‐tumor immunity, such as oncolytic virus therapy (OVT), antibody‐coupled drugs (ADC), bispecific antibodies (BiTEs), have been developed. Although these therapies are not as popular as ICT or adoptive cell transfer therapy due to their mediocre effect and the lack of clinical data, some drugs based on these strategies have been synthesized, and some clinical trials are underway (**Table** **2**).

**Table 2:** A brief summary of anti‐tumor immune drugs and therapies

Type of the therapies	Drug/treatment name	Target	Phase of trial	Indication	Reference
Antibody‐drug conjugate (ADC)	ENHERTU	HER2	Approved	breast cancer, gastric or gastroesophageal junction adenocarcinoma	^[^ ^170^ ^]^
	KADCYLA	HER2	Approved	breast cancer	
	MYLOTARG	CD33	Approved	acute myeloid leukemia	
	ADCETRIS	CD30	Approved	classical Hodgkin lymphoma, systemic anaplastic large cell lymphoma, peripheral T‐cell lymphomas, primary cutaneous anaplastic large cell lymphoma	
	BESPONSA	CD22	Approved	B‐cell precursor acute lymphoblastic leukemia	
	POLIVY	CD79b	Approved	diffuse large B‐cell lymphoma	
	HERCEPTIN HYLECTA	HER2	Approved	breast cancer	N/A
	Sacituzumab govitecan	Trop‐2	Approved	triple‐negative breast cancer	^[^ ^171^ ^]^
Bispecific antibody	Blinatumomab	CD19 and CD3	Approved	B‐cell precursor acute lymphoblastic leukemia	^[^ ^170^ ^]^
	MGD013	LAG‐3 and PD‐1	Phase II/III	gastric cancer, squamous cell carcinoma of head and neck	^[^ ^172^ ^]^
	FS118	LAG‐3 and PD‐L1	Phase I/II	advanced cancer, metastatic cancer, squamous cell carcinoma of head and neck	^[^ ^173^ ^]^
	RO7121661	Tim‐3 and PD‐1	Phase I/II	metastatic melanoma, non‐small cell lung cancer , small cell lung cancer , esophageal squamous cell carcinoma, urothelial carcinoma	^[^ ^174^ ^]^
	MGD009	B7‐H3 and CD3	Phase I	advanced solid tumors	^[^ ^175^ ^]^
	VT1021	CD36 and CD47	Phase I	solid tumor	^[^ ^102^ ^]^
	PRS‐343	4‐1BB and HER2/4	Phase I	breast cancer, gastric cancer, bladder cancer, solid tumor	^[^ ^176^ ^]^
	MCLA‐145	4‐1BB and PD‐L1	Phase I	B‐cell lymphoma, solid tumor	^[^ ^177^ ^]^
	FS120	4‐1BB and OX40	Phase I	advanced tumors	^[^ ^178^ ^]^
	GEN1042	4‐1BB and CD40	Phase I/II	non small cell lung cancer, colorectal cancer, melanoma	N/A
CAR‐T	Kymriah	CD19	Approved	B‐cell precursor acute lymphoblastic leukemia , large B‐cell lymphoma	^[^ ^179^, ^180^ ^]^
	Yescarta	CD19	Approved	large B‐cell lymphoma	
	Brexucabtagene Autoleucel	CD19	Approved	relapsed or refractory mantle cell lymphoma	^[^ ^180^ ^]^
CAR‐NK	iC9/CAR.19/IL15‐Transduced CB‐NK Cells	CD19	Phase I/II	non‐Hodgkin’s lymphoma, chronic lymphocytic leukemia	^[^ ^181^ ^]^
Oncolytic virus	T‐Vec		Approved	melanoma	^[^ ^182^ ^]^
	Telomelysin		Phase II	head and neck squamous cell carcinoma, esophagogastric adenocarcinoma, gastric and gastroesophageal junction adenocarcinoma	^[^ ^183^ ^]^
	CAVATAK		Phase I/II	melanoma, non‐muscle invasive bladder cancer, head and neck cancer, non‐small cell lung cancer	^[^ ^184^ ^]^
	CG0070		Phase II/III	non muscular invasive bladder cancer	^[^ ^185^ ^]^
	Toca511		Phase II/III	glioblastoma multiforme, anaplastic astrocytoma	^[^ ^182^ ^]^
	Reolysin		Phase II	pancreatic adenocarcinoma, breast cancer, osteosarcoma, ewing sarcoma family tumors, malignant fibrous histiocytoma, synovial fibrosarcoma, leiomyosarcoma, prostate cancer	^[^ ^186^ ^]^
AXAL	ADXS11‐001		Phase II/III	squamous cell carcinoma, anal cancer, rectal cancer, cervical cancer	^[^ ^187^ ^]^
	ADXS31‐164		Phase I/II	HER2 expressing solid tumors	^[^ ^188^ ^]^
	ADXS‐NEO		Phase I	solid tumors	^[^ ^189^ ^]^
	ADXS‐503		Phase I/II	non‐small cell lung cancer	^[^ ^190^ ^]^
	ADXS31‐142		Phase I/II	prostate cancer	^[^ ^191^ ^]^
Cancer vaccine	Sipuleucel‐T		Approved	prostate cancer	^[^ ^192^ ^]^
	CimaVax		Phase II/III	non‐small cell lung carcinoma, head and neck squamous cell carcinoma	^[^ ^193^, ^194^ ^]^
	Vitespen		Phase III	melanoma, renal cell carcinoma	^[^ ^195^, ^196^ ^]^
	WT1		Phase I/II	pleural mesothelioma, myeloid malignancies	^[^ ^197^, ^198^ ^]^
	IGV‐001		Phase II	glioblastoma	N/A

### 5.3 Oncolytic Virus Therapy and Bispecific Antibodies

Several viruses like human papilloma virus (HPV) and Epstein‐Barr virus (EBV) have the ability to induce the formation of tumors.^[^ ^199^, ^200^ ^]^ However, there are also viruses that can be used to kill tumor cells. Oncolytic virus (OV) immunotherapy is an anti‐tumor therapy that utilizes native or genetically modified viruses that selectively replicate within tumor cells to kill those tumor cells.^[^ ^201^ ^]^ Besides, oncolytic virus immunotherapy can improved the effect of ICT and therefore benefit patients who are not responsive to ICT monotherapy.^[^ ^202^, ^203^ ^]^ In these years, oncolytic virus immunotherapy have made a great progress, many engineering oncolytic viruses based on different family of viruses have been designed and have entered different stages of clinical trials.^[^ ^182^, ^204^ ^]^ In 2018, Xu et al. reported that the expression of E‐cadherin induced by the virus enhances the transmission and the oncolytic ability of the intratumoral virus.^[^ ^205^ ^]^ Notably, EBV, a virus that can induce the formation of tumor, can also be used to kill EBV‐positive tumors and induce anti‐tumor immunity.^[^ ^206^, ^207^ ^]^ In 2020, Choi et al. discovered LMP1, an important signaling protein that can induce the activation of CD4^+^ T cells and therefore induce the anti‐tumor immunity.^[^ ^207^ ^]^

Another potential strategy is the use of bispecific antibodies, which can simultaneously bind tumor‐specific antigens and some surface molecules on different immune cells to activate immune cells and guide them to kill tumors in multiple ways. At present, various bispecific antibodies have been developed.^[^ ^208^ ^]^ For example, Ruiz et al. synthesized bispecific antibodies targeting HER2 using the carboxyl terminal fragment p95her2 of the receptor as a tumor‐specific antigen. This novel antibody significantly reduced the toxicity and side effects compared with the previous one.^[^ ^209^ ^]^ CD3, an important surface molecule expressed on T cells, is usually used in designing bispecific antibodies. Skokos et al. found that the activation of CD28, another surface molecule on T cells, can promote the anti‐tumor effect of CD3 bispecific antibodies.^[^ ^210^ ^]^ Wu et al. developed three specific antibodies based on this mechanism.^[^ ^211^ ^]^ Recently, Millar et al. developed a novel anti‐tumor method by delivering antigens to the tumor surface using the antibody peptide epitope conjugate (APEC), thus guiding CMV‐specific CD8^+^ T cells for targeted killing.^[^ ^212^ ^]^ Furthermore, bispecific antibodies that target neoantigens using tumor‐specific mutants predicted by computers have been synthesized to directionally kill tumor cells. Those targeting mutant *TP53*, mutant *RAS*, and neoantigens on T cells have shown great effects in mice.^[^ ^213^, ^214^, ^215^ ^]^

### 5.4 Immunotherapies Based on Other Immune Cells Beyond T Cells and B Cells

Besides T cells and B cells, other immune cells are essential in anti‐tumor immunity.^[^ ^216^ ^]^ For example, TAMs, an important group of tumor‐infiltrating immune cells and commonly appear as M2‐like macrophages, inhibit anti‐tumor responses. Thus, depleting or stimulating macrophages have been used to relieve immunosuppression and enhance the anti‐tumor response. In addition to previously identified targets, such as CSF‐1/CSF‐1R, CD40, and CD47, two members of siglec family, siglec‐10 and siglec‐15, mediate pathways that affect phagocytosis and the effector function of CD8^+^ T cells, respectively.^[^ ^82^, ^217^ ^]^ CD73 and Class IIa HDAC are potential macrophage targets recently discovered, and their blockade has been to exert an anti‐tumor response in mice.^[^ ^218^, ^219^ ^]^ Like TAMs, tumor‐associated neutrophils (TANs) is also heterogeneous with different or even opposite functions.^[^ ^200^ ^]^ In 2009, Fridlender et al. revealed that TGF‐*β* may be one of the key molecule to the determination of the anti‐tumor TANs (“N1” phenotype) or immunosuppressive TANs (“N2” phenotype).^[^ ^220^ ^]^ After that, more and more researchers have focused on this field and the opposite functions of TANs have been proved in different cancers.^[^ ^221^, ^222^, ^223^ ^]^ In 2020, Jaillon et al. reviewed the diversity and plasticity of TANs and prospected the future of anti‐tumor therapies based on TANs.^[^ ^224^ ^]^ However, there are still plenty of controversies in this field, some of the researches showed opposite results.^[^ ^225^ ^]^ Therefore, we need more researches and evidence of TANs to know the population better.

Common myeloid progenitor (CMP) is one of the most important stem cells in the bone marrow. They can differentiate into macrophages and neutrophils, thus indirectly affecting anti‐tumor immunity. Two groups of researchers recently developed an attractive strategy based on trained immunity for cancer treatmen, respectively: Kalafati at al. observed inhibition of tumors on mice treated with *β*‐glucan, a prototypical agonist of trained immunity and they then proved*β*‐glucan may induce the transcriptomic and epigenetic rewiring of granulopoiesis and the appearance of anti‐tumor phenotype of TANs.^[^ ^226^ ^]^ Another group of researchers, Priem and his colleagues, developed a bone marrow‐avid nanobiologic platform to induce anti‐tumor trained immunity and obtained satisfactory results in a B16F10 mouse melanoma model.^[^ ^227^ ^]^ We think this strategy is worthy of clinical evaluation.

Another population of cells that differentiate from CMPs is MDSCs. There are two major groups of MDSCs, polymorphonuclear MDSCs (PMN‐MDSCs) and monocytic MDSCs (M‐MDSCs), which mainly suppress the anti‐tumor response of T cells.^[^ ^228^ ^]^ Because of the functions of MDSCs in anti‐tumor immunity, they have been widely used as anti‐tumor targets. The potential of anti‐CD33 antibody, LXR agonist, and ATAR in anti‐tumor treatment has been reported. In 2021, Veglia et al. comprehensively reviewed the characteristics and therapeutic targets of MDSCs.^[^ ^229^ ^]^

APCs are another important group of cells in anti‐tumor immunity. They are highly heterogeneous and play multiple roles in TMEs. Antigen presentation, include tumor antigen presentation, is an important function shared by many APCs while tumors also developed plenty of strategies to escape being recognized by APCs or even to inhibit the activity of APCs.^[^ ^228^ ^]^ In that case, reactivating or increasing APCs may be a potential anti‐tumor strategy. Tumor vaccines may be an effective way to stimulate APCs and active effector T cells to kill tumor cells. Different types of tumor vaccines have been developed, including subunit vaccines, DC‐based vaccines, mRNA vaccines and so on.^[^ ^160^, ^230^ ^]^ A group of scientists linked the vaccine component with albumin to develop a subunit vaccine and got remarkable anti‐tumor effect in mice.^[^ ^220^ ^]^ DC‐based vaccines transfer DC‐loading antigens to activate anti‐tumor immunity, with several clinical trials have been done.^[^ ^231^ ^]^ A recent clinical study has developed a new type of in situ tumor vaccine and reported its great clinical response in combination with PD‐1 in the treatment of indolent non‐Hodgkin's lymphomas.^[^ ^232^ ^]^ Furthermore, personalized vaccines, which means vaccines designed to target unique mutations in a certain patient, may become a new direction owing to new technologies that can rapidly map mutations within the genome. Though there are still many challenges, personalized cancer vaccines have made exciting progress in clinical translation.^[^ ^233^ ^]^ AXAL is another notable strategy that uses the bacterial vector *Listeria monocytogenes* to stimulate APCs and has been used in clinical trials.

NK cells can directly kill tumor cells with low MHC I expression or ADCC. Many strategies based on NK cells to enhance anti‐tumor response have been proposed.^[^ ^234^ ^]^ In 2018, Qing et al. reported that the TIGIT blockade prevents NK cell exhaustion and enhanced the anti‐tumor response.^[^ ^235^ ^]^ In 2020, Myers and Miller reviewed the research progress on NK cells and comprehensively summarized anti‐tumor immunotherapies targeting NK cells. Natural killer T (NKT) cells are a special population of T cells that exhibit the main features of both CD8^+^ T cells and NK cells and have shown remarkable anti‐tumor effects. For instance, a single‐domain antibody (VHH) that can specifically induce strong‐type I NKT cell activation has been developed^[^ ^236^ ^]^ and may play an important role in future anti‐tumor immunotherapies.

Furthermore, adoptive cell transfer therapy infuses highly active autologous or allogeneic immune cells into cancer patients to kill tumor cells. In addition to well‐known therapies based on T cells, such as TIL, TCR‐T, and CAR‐T, several adoptive cell transfer therapies have used other immune cells. CAR‐Ms can decrease the tumor burden by expressing pro‐inflammatory cytokines and chemokines to convert bystander M2‐like macrophages into M1‐like macrophages and many other pathways.^[^ ^237^ ^]^ CAR‐NK cells exert an effective anti‐tumor response with less toxicity than CAR‐T cells.^[^ ^238^ ^]^ To date, many clinical trials employing CAR‐NK cells are underway.^[^ ^239^ ^]^ In a 2020 clinical trial, Liu et al. showed that most patients with relapsed or refractory CD19‐positive cancers responded to treatment with CAR‐NK cells and no major toxic effects were detected.^[^ ^181^ ^]^ In the same year, an initial clinical trial reported that CAR‐NKT cells showed certain effects without strong toxicity, indicating their potential for expansion in the clinical setting.^[^ ^240^ ^]^

### 5.5 Gut Microbiota Influence Anti‐Tumor Therapies

The gut flora has also been associated with the occurrence and treatment of cancers. Yachida et al. observed changes in the gut microbial community composition during the development of colorectal cancer.^[^ ^241^ ^]^ In 2018, Ma et al. found that the gut microbiota induces a liver‐selective anti‐tumor effect through NKT cells,^[^ ^242^ ^]^ suggesting that different immune cells may play a role in the anti‐tumor responses mediated by the gut microbiota. In addition, the importance of the gut microbiota in the efficacy of checkpoint blocking therapy has been demonstrated.^[^ ^243^, ^244^, ^245^, ^246^ ^]^ Therefore, fecal bacteria transplantation can be beneficial as an adjuvant anti‐tumor therapy.

## 6 Results and Discussion

Tumor immunotherapy is gaining increasing attention and is a promising strategy in anti‐tumor immunotherapies. However, many patients do not benefit from traditional PD‐1 or CTLA‐4 blockade. Therefore, it is of utmost importance to explore why immunotherapies fail and identify novel therapeutic targets. CD8^+^ T cells are one of the main cells affected by checkpoint blockade. Many proteins expressed on CD8^+^ T cells can be used as drug targets. Recently, the signaling pathways of many target molecules have been identified. The composition and behavior of CD8^+^ T cells in the TME have also been explored. CD4^+^ T cells are another type of important cell in the TME. Some CD4^+^ T cells release cytokines to promote anti‐tumor response and directly kill tumor cells. However, Treg cells, the most important CD4^+^ T cells, play an immunosuppressive role in the TME. Therefore, inhibiting their activation in the TME without causing adverse effects, such as autoimmunity, has become a research challenge. Most studies have focused on elucidating the mechanism underlying the special functions of Tregs and searching for suitable anti‐tumor targets on Treg cells.

Exhausted T cells are another special population of T cells in the TME. Some studies have focused on the program of the formation ofTex cells, while others have found some important proteins in this program, such as TOX, TCF‐1, T‐bet, and Eomes. The dynamic changes in Tex cells and their function during different stages contribute to further our understanding.

Only a few studies on the role of B cells in the TME have been conducted. Notably, their essential functions in anti‐tumor immunity have been revealed. These functions are mediated not only by the antibodies released by B cells but also by several other pathways. The antigen‐presenting function of B cells, ADCC mediated by B cells, the function of exosomes released by B cells, and some other functions of B cells in the TME have been reported. Future studies should focus on B cells.

Finally, we introduce novel anti‐tumor treatment strategies based on various immune cells in the TME, some of which have strong targeting effects and can replace traditional tumor therapies, which have poor efficacies and strong adverse effects. Other novel strategies may complement ICT and adoptive cell transfer therapy and thus be beneficial for patients nonresponsive to these two immunotherapies. It is also important to comprehensively understand the different immune cells in TMEs. Several of these have become targets of novel treatments, whereas others may become the next focus of antitumor therapies.

Combination therapies have promising potential as cancer treatment. Currently, it is a widely accepted strategy to transform “cold” tumors into “hot” tumors. The combination of some drugs and methods often achieves enhanced effects in eliminating tumors.

Understanding the features of different TME in real time using new technologies, such as single‐cell sequencing, is vital in tumor immune research as the TME has high heterogeneity. Recently, the landscapes of lung cancer, liver cancer, colon cancer, and other cancers have been investigated.^[^ ^247^, ^248^, ^249^, ^250^, ^251^, ^252^, ^253^ ^]^ These studies have revealed the composition of tumor‐infiltrating cells in different tumors and identified closely related molecules to immunotherapies. All these studies provide a further understanding of different TMEs. It can be predicted that with the development of new technologies such as single‐cell sequencing, they will be increasingly used in tumor immune research and greatly accelerate the research process in the studies of antitumor immunity.

Many of the potential target proteins mentioned in this paper have evaluated as targets in clinical trials, and other targets are underway. Increased understanding of the maturity of immunotherapies, we believe more effective methods to treat cancers can be soon discovered. However, the mechanisms by which some immune cells function in anti‐tumor immunity and their corresponding pathways remain unknown. Therefore, future should explore these immune cells and the targeted proteins associated with anti‐tumor immunity.

## Conflict of Interest

The authors declare no conflict of interest.

## Author Contributions

Y.X., F.X., and L.Z. contributed equally to this work. Y.X., F.X., and L.Z. conceived and drafted the manuscript, drew the figures, and summarized the tables. Y.X., F.X., L.Z., X.Z., J.H., F.W., J.J., L.Z., and F.Z. discussed the concepts of the manuscript. F.Z. approved the version to be submitted.
